# Restrictive versus high-dose oxygenation strategy in post-arrest management following adult non-traumatic cardiac arrest: a meta-analysis

**DOI:** 10.1186/s13054-023-04669-2

**Published:** 2023-10-05

**Authors:** S. Macherey-Meyer, S. Heyne, M. M. Meertens, S. Braumann, C. Hueser, V. Mauri, S. Baldus, S. Lee, C. Adler

**Affiliations:** 1 Faculty of Medicine and University Hospital Cologne, Clinic III for Internal Medicine, University of Cologne, Kerpener Straße 62, 50937 Cologne, Germany; 2 Center of Cardiology, Cardiology III -Angiology, University Medical Center of the Johannes Gutenberg-University, Mainz, Germany; 3 Faculty of Medicine and University Hospital Cologne, Clinic II for Internal Medicine, University of Cologne, Cologne, Germany; 4 Faculty of Medicine and University Hospital Cologne, Emergency Department, University of Cologne, Cologne, Germany

**Keywords:** Cardiac arrest, OHCA, Oxygenation, Oxygen saturation, Hypoxemia, Hyperoxemia

## Abstract

**Purpose:**

Neurological damage is the main cause of death or withdrawal of care in comatose survivors of cardiac arrest (CA). Hypoxemia and hyperoxemia following CA were described as potentially harmful, but reports were inconsistent. Current guidelines lack specific oxygen targets after return of spontaneous circulation (ROSC).

**Objectives:**

The current meta-analysis assessed the effects of restrictive compared to high-dose oxygenation strategy in survivors of CA.

**Methods:**

A structured literature search was performed. Randomized controlled trials (RCTs) comparing two competing oxygenation strategies in post-ROSC management after CA were eligible. The primary end point was short-term survival (≤ 90 days). The meta-analysis was prospectively registered in PROSPERO database (CRD42023444513).

**Results:**

Eight RCTs enrolling 1941 patients were eligible. Restrictive oxygenation was applied to 964 patients, high-dose regimens were used in 977 participants. Short-term survival rate was 55.7% in restrictive and 56% in high-dose oxygenation group (8 trials, RR 0.99, 95% CI 0.90 to 1.10, *P* = 0.90, I^2^ = 18%, no difference). No evidence for a difference was detected in survival to hospital discharge (5 trials, RR 0.98, 95% CI 0.79 to 1.21, *P* = 0.84, I^2^ = 32%). Episodes of hypoxemia more frequently occurred in restrictive oxygenation group (4 trials, RR 2.06, 95% CI 1.47 to 2.89, *P* = 0.004, I^2^ = 13%).

**Conclusion:**

Restrictive and high-dose oxygenation strategy following CA did not result in differences in short-term or in-hospital survival. Restrictive oxygenation strategy may increase episodes of hypoxemia, even with restrictive oxygenation targets exceeding intended saturation levels, but the clinical relevance is unknown. There is still a wide gap in the evidence of optimized oxygenation in post-ROSC management and specific targets cannot be concluded from the current evidence.

**Supplementary Information:**

The online version contains supplementary material available at 10.1186/s13054-023-04669-2.

## Introduction

Cardiac arrest (CA) can be dichotomized between out-of-hospital cardiac arrest (OHCA) and in-hospital cardiac arrest (IHCA) [1, 2]. The prevalence of CA increases: OHCA affects 67 to 170 per 100.000 Europeans per year [1, 3, 4]; while, IHCA is documented in 1 to 7 cases per 1000 patients yearly [1, 5]. IHCA is associated with a better prognosis than OHCA. In OHCA only 7–11% of patients survive until hospital discharge. Of these, only few have a favorable neurological outcome with full recovery or disabilities compatible with independent daily living [2–4, 6, 7]. Patients with CA are vulnerable and require all amendable efforts to strengthen the chain of survival [8]. Cardiopulmonary resuscitation (CPR) is mandatory to maintain blood flow and concomitantly highest possible inspired oxygen concentration is recommend during chest compression [8].

Irreversible and diffuse neurological damage is the main cause of death after CA. Adequate oxygen delivery to the brain is key for the preservation of neuronal homeostasis and individual nerve cell survival [9, 10]. ILCOR pragmatically recommends 100% inspired oxygen after return of spontaneous circulation (ROSC) until first blood gas analysis, but precise subsequent oxygen targets are not defined [8]. New studies (HOT-ICU, BOX, EXACT) have been published after the latest, "neutral" ILCOR recommendations for post-ROSC oxygenation [11–14]. The results of these studies could potentially further inform postresuscitation practice. Following the emergence of this new evidence, a meta-analysis is warranted, in order to further elucidate the efficacy of restrictive vs. high-dose oxygenation strategies.

## Material and methods

This meta-analysis was conducted using a pre-specified protocol and reproducible plan for literature search and synthesis according to the Preferred Reporting Items for Systematic reviews and Meta-Analysis (PRISMA) guidelines [15]. The meta-analysis was prospectively registered in PROSPERO database (CRD42023444513). The systematic literature search was performed in three data bases including Medline (via PubMed), Web of Science and Cochrane Library. The search strategy for each database is provided in the supplementary appendix. The search was performed on July 12th 2023. No restrictions on publication date, language or study size were applied. After exclusion of duplicates and screening of titles and abstracts according to the eligibility criteria, full-texts of the remaining articles were assessed.

The study selection was independently performed by two reviewers (SM, MMM). In case of any disagreement, this was resolved by consensus with one of the senior authors (SL/CA).

Randomized controlled trials (RCT) comparing two competing oxygenation strategies in post-ROSC management of patients with CA were eligible. Oxygenation strategies should follow a lower (“restrictive”, intervention group) and higher dose (control group) regimen. We did not define explicit thresholds for this review. Start of intervention was applicable in all settings including preclinical and in-hospital periods. No restrictions were applied for follow-up duration or duration of intervention itself. Double publications, cluster- or pseudo-randomized studies, case reports, case series without control groups, reviews and conference abstracts were excluded.

Data were extracted by one investigator (SMM) using a standardized pre-specified data collection form. Main study reports as well as any supplementary appendices and study protocols were reviewed. Pre-specified data elements included study design, patient baseline characteristics, intervention and follow-up data.

The primary efficacy end point was short-term survival defined as overall survival within 90 days after CA. Within the 90-day range the longest reported follow-up of each trial (e.g. in-hospital, 30-day and 90-day survival) was eligible and extracted for quantitative analysis. Secondary efficacy outcomes were survival to hospital discharge, survival to intensive care unit (ICU) discharge and favorable neurological outcome at discharge. The latter was defined by cerebral performance category score ≤ 2. Safety outcome was the number of patients with episodes of hypoxemia. As these are not generally defined, we considered all desaturations of oxygen saturation < 90%.

Risk of bias at study level was assessed using the Cochrane Collaborations risk-of-bias tool (RoB2, version 08/22/2019) for randomized trials [16]. Risk of bias assessment was performed by two individual investigators (SMM, SH). In case of discrepancy a third independent investigator was consulted (MMM). Risk of bias assessment was performed regarding the prioritized outcome short-term survival.

Random-effects meta-analyses were performed using the Mantel–Haenszel method for dichotomous event data. Pooled risk ratios (RRs) and 95% confidence intervals (CI) are given for each analysis with a two-sided significance level of *P* < 0.05 (RevMan 5.3, Nordic Cochrane Centre, Cochrane Collaboration). The extent of heterogeneity was approximated by I^2^ tests considering 0–40% as non-important, 30–60% as moderate, 50–90% as substantial and 75–100% as considerable heterogeneity. Pre-specified analysis of publication bias by funnel plot was not appropriately feasible given the low number of studies included.

Post-hoc sensitivity meta-analysis of primary outcome was performed according to risk of bias judgement. RCTs at “high” risk of overall bias were excluded. Post-hoc subgroup meta-analysis of primary outcome was performed according to oxygenation targets, and timing of intervention (pre-hospital intervention). Meta-regression was preliminarily planned by protocol, but was cancelled given the interstudy heterogeneity in design and considering concerns about the certainty of measured effects on individual trial level.

We did not obtain ethical approval for this meta-analysis because we did not collect data from individual human subjects.

## Results

### Study selection

**Fig. 1:**
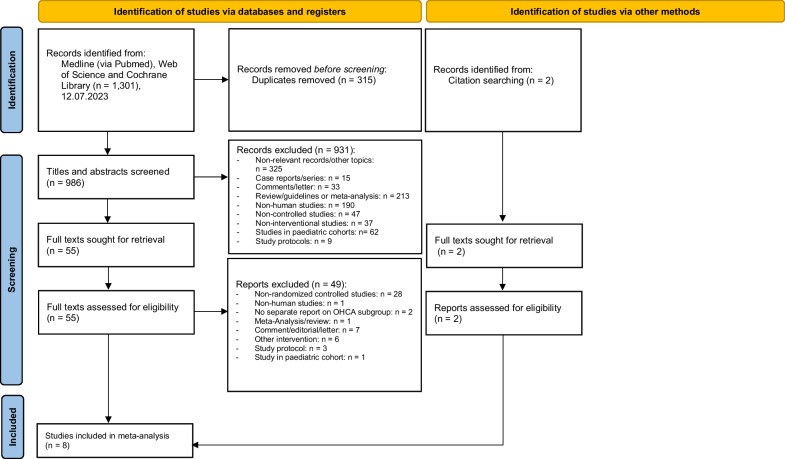
Flowchart diagram

A total of 1,301 articles were identified by the described search strategy (see Fig. 1, PRISMA Flow chart). After removing duplicates, the titles and abstracts of 986 remaining articles were screened. 931 articles were excluded which left 55 references for assessment of full-text eligibility. Two additional full-texts were assessed for eligibility by handsearching. Eight studies were finally included in quantitative analyses.

### Studies

Eight RCTs were included in meta-analysis (see Table 1) [11, 13, 14, 17–21]. Six trials enrolled OHCA patients, and two trials included both IHCA and OHCA [11, 21]. Three trials were designed as feasibility or pilot studies [17–19]. In four trials study treatment was established during preclinical course [14, 17–19], and in four studies patients were enrolled and treated after arrival at the emergency department (ED) or ICU [11, 13, 20, 21]. All but three trials enrolled solely patients with CA from assumed cardiac cause [13, 14, 18–20]. One trial solely included patients with witnessed CA [17], and in four trials shockable rhythm was required for inclusion [17–20]. Three trials precisely defined oxygen targets based on oxygen saturation measured in blood gas analyses and oxygenation strategy had to be adjusted to measurements per protocol [11, 13, 20]. In four trials a titrated oxygen delivery was defined as interventional strategy, and two investigator groups subsequently defined precise SpO2 (oxygenation saturation measured in pulse oximetry) targets [14, 18, 19, 21]. In one feasibility trial patients were randomized to two different FiO2 (fraction of inspired oxygen) levels without a specified target saturation [17]. Duration of study intervention was restricted to preclinical treatment period in two trials [14, 19], and limited to 60 min after ROSC in one trial [17]. One trial offered study treatment for up to a 90-day treatment period [11].

**Table 1:** Characteristics of included studies and patients

		Kuisma et al. (2006)	HOT or NOT 2014	EXACT PILOT 2018	COMACARE 2018	ICU-ROX substudy 2020	BOX 2022	EXACT 2022	HOT-ICU substudy 2023
Study characteristics									
Study period and location		-,Finland	10/2012–09/2013, New Zealand	07/2015–05/2017 Australia	03/2016–11/2017 Finland, Denmark	09/2015–05/2018 Australia, New Zealand	03/2017–12/2021 Denmark	12/2017–08/2020 Australia	06/2017–08/2020, international trial
Study design		RCT, multi-centric, single-blind design	RCT, multi-centric, single-blind design	RCT, multi-centric, single-blind design	RCT, multi-centric, single-blind design	RCT, multi-centric, single-blind design	RCT, multi-centric, single-blind design	RCT, multi-centric, single-blind design	RCT, multi-centric, single-blind design
Interventional strategy “Restrictive”		FiO2 30% for 60 min after ROSC, manual ventilation	Titrated oxygen therapy at mechanical ventilation, Sp02 90–94% or Fi02 0.4, until 72 h after randomization or extubation; start on scene	Titrated oxygen therapy 2L/min (4L/min at one study site), application through bag-valve reservoir	Normoxia, 10-15 kPa delivered by invasive mechanical ventilation, for 36 h after ICU arrival or until extubation or until spontaneous breathing, SpO2 target 95–98%	Titrated oxygen therapy aiming at FiO2 21%, lower SpO2 alarm at 90%, upper SpO2 alarm at 97%, treatment until ICU discharge or 28 days from randomization	PaO2 of 9–10 kPa delivered by invasive mechanical ventilation	Titrated oxygen therapy with target SpO2 90–94%, application through bag-valve reservoir 4L/min or mechanical ventilation (FiO2 0.6) until hospital arrival	PaO2 of 8 kPa delivered by invasive or non-invasive mechanical ventilation, up to 90 days
Comparator treatment “High-dose”		FiO2 100% for 60 min after ROSC, manual ventilation	Standard oxygen therapy at mechanical ventilation, Sp02 > 95%, until 72 h after randomization or extubation; start on scene	Standard oxygen therapy 10L/min (4L/min at one study site), application through bag-valve reservoir	Hyperoxia, 20-25 kPa delivered by invasive mechanical ventilation, for 36 h after ICU arrival or until extubation or until spontaneous breathing,	Standard oxygen therapy, lower SpO2 alarm at 90%, no upper SpO2 alarm. FiO2 < 30% was discouraged, treatment until ICU discharge or 28 days from randomization	PaO2 of 13–14 kPa delivered by invasive mechanical ventilation	Titrated oxygen therapy with target SpO2 98–100%, application through bag-valve reservoir 10L/min or mechanical ventilation (FiO2 1.0) until hospital arrival	PaO2 of 12 kPa delivered by invasive or non-invasive mechanical ventilation, up to 90 days
Start of intervention		Prehospital setting	Prehospital setting	Prehospital setting	ICU setting	ICU setting	ICU setting/ED	Prehospital setting	ICU setting
Number of patients enrolled		32	18	62	123	166	802	425	335
Cohort		Witnessed OHCA with VF	OHCA with presumed cardiac cause and VT/VF arrest	OHCA with presumed cardiac cause and VT/VF arrest	OHCA with presumed cardiac cause and VT/VF arrest	OHCA/IHCA from any origin and suspected hypoxic ischaemic encephalopathy	OHCA with presumed cardiac cause	OHCA with presumed cardiac cause	OHCA or IHCA from any origin
TTM		At treating physician’s discretion	Mandatory therapeutic hypothermia	At treating physician’s discretion	Mandatory TTM at either 33 °C or 36 °C	At treating physician’s discretion	Mandatory TTM at 36 °C	At treating physician’s discretion	-
Primary outcome		Survival to hospital discharge	Sp02 in pre-hospital period	Sp02 ≥ 94% at hospital arrival, proportion of patients	NSE serum concentration	Composite: death or unfavorable neurological outcome at 180 days	Composite: death or CPC score > 2	Survival to hospital discharge	All-cause mortality at 90 days
Main secondary outcomes		Blood gases, Need to raise FiO2, Biomarker testings	SpO2 serially measured, PaO2, Hypoxemic episodes (Sp02 < 88%), survival to hospital discharge	Sp02 ≥ 90% at hospital arrival, Re-arrest, Survival to hospital discharge	Blood gases, Biomarker concentrations, Regional fronto cerebral oxygenation, EEG, favorable neurological outcome (CPC score ≤ 2) at 6 months, ICU length of stay, hospital length of stay, 30-day survival, serious adverse events	Mortality: ICU, in-hospital, 180 days, ICU length of stay, hospital length of stay, ventilator-free days, vasopressor-free days	Time to death, Discharge with CPC score > 2, vasopressor use, Biomarker testings, cognitive assessment, adverse events	Re-arrest before ICU arrival, Hypoxemia (< 90%) before ICU arrival, Survival to ICU discharge, ICU length of stay, Hospital length of stay, favorable neurological outcome (CPC score ≤ 2) at hospital discharge, SAE 12 months survival 12 months QoL	Days alive without life support, Days alive and out of hospital, SAE at ICU 1-year survival QoL
Baseline characteristics of patients included									
Restrictive group	Patients	14	8	37	61	87	394	214	149
High-dose group	Patients	14	9	24	59	79	395	211	186
Restrictive group	Age, median	61.9 (13.6)	71.6 (10.7) Mean	64 (13.5) Mean	59 (13) Mean	62.3 (14.8) Mean	62 (13)	66.4	70
High-dose group	Age, median	64.3 (7.8)	61.4 (20.8) Mean, SD	60.5 (9) Mean, SD	60 (14) Mean	60.6 (16.1) Mean	63 (14)	64.2	71
Restrictive group	Male patients	13/14	7/8	32/37	50/61	66/87	325/394	163/214	92/149
High-dose group	Male patients	10/14	9/9	17/24	48/59	62/79	312/395	162/211	138/186
Restrictive group	Shockable rhythm	14/14	8/8	37/37	61/61	58/87	334/393	128/214	–
High-dose group	Shockable rhythm	14/14	9/9	24/24	59/59	46/79	333/394	135/211	–
Restrictive group	Witnessed CA	14/14	6/8	27/37	61/61	72/87	333/394	166/214	–
High-dose group	Witnessed CA	14/14	6/9	18/24	59/59	61/79	339/394	169/211	–
Restrictive group	Bystander CPR	10/14	6/8	31/37	50/61	65/87	346/388	163/214	–
High-dose group	Bystander CPR	6/14	7/9	23/24	48/59	56/79	333/388	157/211	–
Restrictive group	Collapse to ROSC, min, median	17.4 (5.8)	28.6 (12.6)	18.5	20	26.5 (17.8) Mean	21 (13)	27.0	–
High-dose group	Collapse to ROSC, min, median	20.4 (5.7)	30.8 (16.3)	19.5	21	25.4 (14.7) Mean	21 (14)	25.0	–
Restrictive group	Invasive mechanical ventilation	–	–	–	61/61	87	394/394	157/214	142/149
High-dose group	Invasive mechanical ventilation	–	–	–	59/59	79	395/395	159/211	175/186
Restrictive group	Vasopressors	7/14	–	–	–	33/87	359/394	–	11/149
High-dose group	Vasopressors	7/14	–	–	–	34/79	367/395	–	11/186
Restrictive group	TTM	6/14	8/8	–	61/61	72/87	394/394	190/214	–
High-dose group	TTM	8/14	8/9	–	59/59	65/79	395/395	186/211	–

*FiO2* fraction of inspired oxygen, *PaO2* partial pressure of arterial oxygen, *SaO2* oxygen saturation, *CA* cardiac arrest, *CPC* cerebral performance category, *CPR* cardiopulmonary resuscitation, *ED* emergency department, *EEG* electroencephalogram, *ICU* intensive care unit, *OHCA* out-of-hospital cardiac arrest, *RCT* randomized controlled trial, *ROSC* return of spontaneous circulation, *SAE* serious adverse event, *TTM* targeted temperature management, *QoL* quality of life

### Assessment of bias

Assessment and judgement of bias were performed by two investigators (see Table 2) [16]. Four trials were judged to be at “high” risk of overall bias [14, 17–19]. This judgement was mainly driven by serious confounding in the “deviations from intended intervention” domain. In detail, in three trials the restrictive oxygenation group had higher median oxygen saturation than defined per protocol. This raised concerns about substantial performance bias. In the HOT or NOT trial there was a wide overlap of SpO2 curves and no separation of oxygenation curves in preclinical course [18]. This resulted in concerns on the protocol adherence. In the EXACT PILOT trial the titration strategy was changed within the study period, but no analysis to estimate the effect of adhering was performed [19]. Two of these trials were prematurely stopped, one regarding safety concerns of intervention and the other due to COVID 19 pandemic [14, 19]. Two trials with small patient numbers had imbalances in baseline characteristics, and did not provide sufficient information on randomization process [17, 19]. We judged this to be at high risk of bias in “randomization” and consequently in “overall” domain.

**Table 2:** Risk of bias assessment of randomized controlled trials

Risk of bias	Randomization	Deviations from intended interventions	Missing outcome data	Measurement of the outcomes	Selection of the reported results	Overall risk of bias
Kuisma et al	High	Some concerns	Some concerns	Low	Some concerns	High
HOT or NOT	Low	High	Low	Low	Some concerns	High
EXACT PILOT	High	High	Low	Low	Some concerns	High
COMACARE	Low	Some concerns	Some concerns	Low	Some concerns	Some concerns
ICU-ROX	Low	Some concerns	Some concerns	Low	Low	Some concerns
BOX	Low	Some concerns	Low	Low	Low	Some concerns
EXACT	Low	High	Low	Low	Low	High
HOT-ICU	Low	Some concerns	Low	Low	Low	Some concerns

In BOX and HOT-ICU trial the patients in interventional group did exceed defined oxygenation targets, but reasons were reported and were consistent with real-world setting [11, 13]. In both trials a substantial number of patients were spontaneously breathing and/or did not require additional oxygen supplementation. In HOT-ICU, the PaO2 target was reached within the first 10 days. In final consideration, BOX and HOT-ICU were judged to raise “some concerns” in “deviations from intended intervention” and “overall” domain.

Two trials-COMACARE and ICU-ROX—were each associated with “some concerns” in overall judgment. This was mainly driven by few missing outcome data and negligible deviations from protocol in measured outcomes [20, 21].

### Patient level baseline characteristics and oxygenation data

A total of 1,941 patients with CA were included. Baseline characteristics are summarized in Table 1. The median age ranged from 59 to 71.6 years, and 77.5% of patients were male. Cardiac arrest was witnessed in 83.8% and bystander CPR was performed in 81.7%. Shockable rhythm was initially documented in 78.6%. Downtime-defined as interval from collapse to ROSC-ranged from 17.4 to 30.8 min. The majority of patients required vasopressors during observation and were treated with targeted temperature management (TTM).

Oxygenation was measured and expressed by various parameters. Available data are summarized in supplementary appendix (see Additional file 1: Table S1). SaO2 (oxygen saturation measured in blood gas analysis), SpO2 and FiO2 were used. None was consistently applied throughout the trials. Hence, statistical comparisons of oxygenation levels at baseline or during treatment were not applicable.

### Primary outcome analysis

**Fig. 2:**
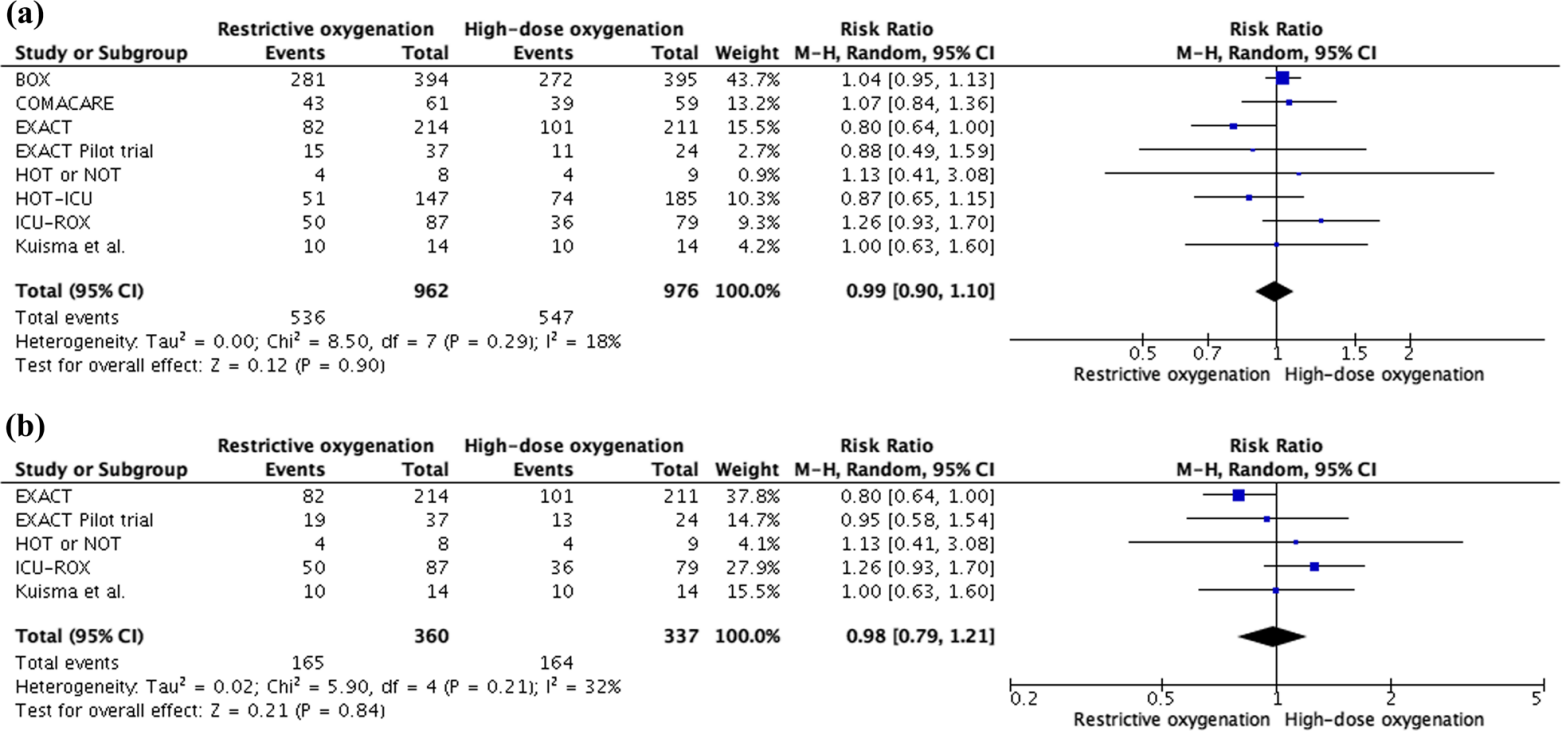
a Short-term survival. **b** Survival to hospital discharge

All eight trials were included in analysis of short-term survival. With respect to missing data 1938 patients were considered. Short-term survival rate was 55.7% in restrictive oxygenation and 56% in high-dose oxygenation group (see Fig. 2a, RR 0.99, 95% CI 0.90 to 1.10, *P* = 0.90, I^2^ = 18%, non-important heterogeneity).

### Secondary efficacy and safety outcome analyses

#### Survival to hospital discharge

Five trials were included [14, 17–19, 21]. The event occurred in 165 participants (45.8%) in restrictive oxygenation and 164 patients (48.7%) in high-dose oxygenation group (see Fig. 2b, RR 0.98, 95% CI 0.79 to 1.21, *P* = 0.84, I^2^ = 32%, non-important heterogeneity).

#### Survival to ICU discharge

Two trials were eligible for analysis [14, 21]. The number of events was 152 (54.5%) and 148 (53.6%) in treatment groups (see Additional file 1: Fig. S1, see Additional file 1: Table S2, RR 1.04, 95% CI 0.81 to 1.35, *P* = 0.74, I^2^ = 61%, substantial heterogeneity).

#### Favorable neurological outcome at discharge

Merely EXACT trial was eligible for analysis [14]. The number of events was 78 (36.6%) and 88 (41.7%) in treatment groups (see Additional file 1: Fig. S2, see Additional file 1: Table S2, RR 0.88, 95% CI 0.69 to 1.11, *P* = 0.28).

#### Episodes of hypoxemia

**Fig. 3:**
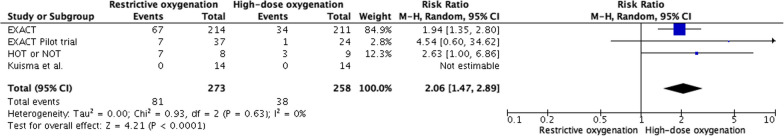
Episodes of hypoxemia

Four trials were included [14, 17–19]. Episodes of hypoxemia occurred in 81 participants (29.7%) in restrictive oxygenation and 38 patients (14.7%) in high-dose oxygenation group (see Fig. 3, RR 2.06, 95% CI 1.47 to 2.89, *P* = 0.004, I^2^ = 13%, non-important heterogeneity, favoring high-dose group).

#### Subgroup and sensitivity analyses

Considering the normoxic target in interventional group of COMACARE (10-15 kPa), this trial was excluded from subgroup analysis [20]. There was no statistically significant difference between the groups in short-term survival (see Additional file 1: Fig. S3, RR 0.98, 95% CI 0.87 to 1.1, *P* = 0.72, I^2^ = 27%, non-important heterogeneity). Evaluation of preclinical initiation of study treatment was performed including four trials [14, 17–19]. There was no statistically significant difference between the groups in short-term survival within the preclinical trials (see Additional file 1: Fig. S4, RR 0.86, 95% CI 0.71 to 1.03, *P* = 0.10, I^2^ = 0%, no heterogeneity).

Four trials were eligible for sensitivity analysis according to RoB assessment [11, 13, 20, 21]. There was no statistically significant difference between the groups in short-term survival (see Additional file 1: Fig. S5, RR 1.04, 95% CI 0.95 to 1.14, *P* = 0.40, I^2^ = 7%, non-important heterogeneity).

## Discussion

Comparison of restrictive and high-dose oxygenation strategy in survivors of CA showed the following novel findings.

The use of restrictive oxygenation strategy may result in no difference in short-term survival or survival to hospital discharge. Then, it may result in a twofold increase in hypoxemic episodes. Finally, the evidence is very uncertain about the effect on survival to ICU discharge or favorable neurological outcome at discharge.

In survivors of CA neurological damage and injury are the main cause of death or withdrawal of care [9, 10]. Hence, post-ROSC management prioritizes neuroprotection. Targeted temperature management was described as an important cornerstone in neuroprotection [22–24], but recent results were controversial and the intervention is discussed intensively [25]. Adequate oxygen delivery to the brain is key for the preservation of neuronal homeostasis and individual nerve cell survival. But specific oxygen targets are missing and hyper- and hypoxemia are potential risks.

Hyperoxemia results in overproduction of reactive oxygen species (ROS) and consequently exacerbates mitochondrial function on molecular and cellular basis [26–29]. In animal studies, hyperoxemia decreased neurological outcome compared to restrictive oxygenation strategy [30]. The clinical effect of hyperoxemia on survivors of CA mainly arises from observational data. Results are inconsistent indicating worse survival [31–33] or no detectable effect compared to normoxemia [34–36]. Notably, these studies considered a wide variance in definitions of hyperoxia ranging from 10 kPa to > 40 kPa. COMACARE provided high quality evidence and did not find differences between normoxemia and hyperoxemia (target 20-25 kPa) in survival analysis [20].

Hypoxemia is discussed to be the main cause of brain injury and hypoxic ischemic encephalopathy, and is supposed to reduce survival after CA [32, 37]. This survival disadvantage was not replicable within the presented trial level data despite the higher number of episodes of hypoxemia. The studies did not specify on the duration or the extent of hypoxemic episodes. The pure number of episodes of hypoxemia does not reflect the effect on clinical outcome. Instead, more promptly treated mild desaturations might be less harmful than a single severe, sustained desaturation. Given the neutral effect of intervention, the clinical implications of this finding on hypoxemic episodes remain unclear. The neutral effect of primary outcome analysis was robust in both subgroup and sensitivity analyses. Interstudy heterogeneity needs to be acknowledged and measured effects should be interpreted with caution.

Included studies used widespread oxygenation strategies and different protocols. On trial level, the intervention itself varied throughout in various dimensions: Start of intervention (preclinical, ED, ICU), application (manual, non-invasive or mechanical ventilation), duration of intervention (60 min to 90 days), treatment strategy (titration, specific dose), target of intervention (SpO2, PaO2, no target) were each heterogeneously performed.

On patient level, the data arise from highly selected cohorts with a considerable proportion of CA from cardiac origin and a high percentage of shockable rhythm. The majority of patients had OHCA, IHCA is underrepresented within the current analysis. A high proportion of patients were male and were treated with bystander CPR. These are well known predictors for favorable outcome [4, 6, 7, 38, 39], and these predictors had a remarkably high prevalence in the studies included compared to unselected OHCA cohorts [1, 4, 5]. These are potential explanations for the remarkable short-term survival rates (55.7% and 56%). Additionally, the design of primary outcome itself inherently underestimates the mortality as four trials merely reported in-hospital survival and information on longer follow-up was not available [14, 17–19].

The inconsistent effects of hyper- and hypoxemia on survival on trial level are well known from general ICU cohorts. Neither hyperoxemia, nor hypoxemia led to differences in overall survival in ICU cohorts in RCTs [12, 40–42]. In final consideration, the authors cannot conclude an optimized oxygenation strategy from the current evidence, but avoidance of both hyper- and hypoxemia seems to be a reasonable approach [8, 43]. The quintessence in study interpretation is the definition of and strategy to reach restrictive oxygenation targets [11, 13, 14]. Single-blinded design, reduced protocol adherence, logistics especially in preclinical period and spontaneously breathing patients not requiring oxygen support might be major contributing factors. Moreover, in the absence of structural pulmonary diseases or ventilation disorders FiO2 of 21% might be sufficient to exceed oxygenation targets. But this limitation in reaching restrictive targets is not a specific phenomenon in the CA cohort. Instead, comparably designed trials enrolling critically ill ICU patients did not reach oxygenation targets in both directions, either [40, 42]. A future trial evaluating the optimal oxygenation strategy in these vulnerable CA patients should acknowledge these barriers in study design.

The current meta-analysis demonstrated comparable survival data in survivors from CA irrespective of restrictive or high-dose oxygenation targets. In contrast, a prior analysis not considering the recently published RCTs found a survival advantage favoring higher dose oxygenation strategies [44]. Consequently, the current meta-analysis adds robust and important evidence. But these results arise from a low level of certainty and have hypothesis-generating implications. The potential effect of restrictive oxygenation on survival data might even be underestimated because a relevant number of patients did not reach intended saturation levels. One might speculate whether more aggressive restriction is clinically reasonable, as episodes of hypoxemia had a significantly higher incidence in these patients. The expected results from ORI-ONE (NCT03653325) and LOGICAL (ACTRN12621000518864) trial might add further evidence to the research question of optimized oxygenation in CA survivors.

### Limitations and strengths

Confounders on individual study level and interstudy heterogeneity were acknowledged. The intervention itself varied throughout the trials and the highly selected cohort each restrict generalizability. The single-blind design might have contributed to failure in reaching oxygen targets. This is a major concern in interpretation and was acknowledged previously [45]. Both subgroup and sensitivity analyses were performed to address these sources of bias. The heterogeneity in outcome definition especially in survival rates (range: in-hospital data to 90 days) limits transferability, too. As four studies only provided in-hospital data, mortality of this cohort is underestimated. Meta-regression was preliminarily planned by protocol to consider variations in oxygenation strategy or targets. But, it was canceled given the interstudy heterogeneity and considering concerns about the certainty of measured effects after risk of bias assessment.

The important strengths of this meta-analysis are the systematic description and discussion of bias, and the adjusted analysis of best available data on oxygenation targets in CA survivors.

## Conclusions

Restrictive and high-dose oxygenation strategy following CA did not result in differences in short-term or in-hospital survival. Restrictive oxygenation strategy may result in a twofold increase in episodes of hypoxemia, even with restrictive oxygenation targets exceeding intended saturation levels, but the clinical implications of this finding are unclear. From the current data an optimal oxygenation strategy or target cannot be concluded for survivors of CA. There is still a wide gap in the evidence of optimized oxygenation in post-ROSC management.

### Supplementary Information

**Additional file 1**: **Fig. S1**. Survival to ICU discharge. **Fig. S2**. Favorable neurological outcome at discharge. **Fig. S3**. Subgroup analysis of primary outcome: oxygenation targets. **Fig. S4**. Subgroup analysis of primary outcome: pre-hospital trials. **Fig. S5**. Sensitivity analysis of primary outcome. **Table S1**. Oxygenation status on trial level. **Table S2**. Event data on efficacy and safety outcomes.

## Data Availability

Data are available and can be extracted from studies included.

## Abbreviations

CA: Cardiac arrest
CI: Confidence interval
CPR: Cardiopulmonary resuscitation
ED: Emergency department
FiO2: Fraction of inspired oxygen
ICU: Intensive care unit
OHCA: Out-of-hospital cardiac arrest
RR: Risk ratio
PaO2: Partial pressure of arterial oxygen
RCT: Randomized controlled trial
ROS: Reactive oxygen species
ROSC: Return of spontaneous circulation
SaO2: Oxygen saturation measured in blood gas analysis
SpO2: Oxygenation saturation measured in pulse oximetry
TTM: Targeted temperature management
