# Supplementary material for: Restrictive versus high-dose oxygenation strategy in post-arrest management following adult non-traumatic cardiac arrest: a meta-analysis
Source: Crit Care. 2023 Oct 5;27:387. doi: 10.1186/s13054-023-04669-2 (PMC10557287; doi:10.1186/s13054-023-04669-2)
Supplement: Supplementary file 1 — Additional file 1: Fig. S1. Survival to ICU discharge. Fig. S2. Favorable neurological outcome at discharge. Fig. S3. Subgroup analysis of primary outcome: oxygenation targets. Fig. S4. Subgroup analysis of primary outcome: pre-hospital trials. Fig. S5. Sensitivity analysis of primary outcome. Table S1. Oxygenation status on trial level. Table S2. Event data on efficacy and safety outcomes. [file 13054_2023_4669_MOESM1_ESM.docx]

**Search details**

*Medline via Pubmed*

("hyperoxia"[MeSH Terms] OR "hyperoxia"[All Fields] OR "hyperoxemia"[All Fields] OR ("hyperoxygenated"[All Fields] OR "hyperoxygenation"[All Fields]) OR "overoxygenation"[All Fields] OR "normoxia"[All Fields] OR "normoxemia"[All Fields] OR (("permissive"[All Fields] OR "permissively"[All Fields] OR "permissiveness"[MeSH Terms] OR "permissiveness"[All Fields] OR "permissivenesses"[All Fields] OR "permissivity"[All Fields]) AND ("hypoxia"[MeSH Terms] OR "hypoxia"[All Fields] OR "hypoxia s"[All Fields] OR "hypoxias"[All Fields])) OR (("permissive"[All Fields] OR "permissively"[All Fields] OR "permissiveness"[MeSH Terms] OR "permissiveness"[All Fields] OR "permissivenesses"[All Fields] OR "permissivity"[All Fields]) AND ("hypoxaemia"[All Fields] OR "hypoxia"[MeSH Terms] OR "hypoxia"[All Fields] OR "hypoxemia"[All Fields] OR "hypoxemias"[All Fields])) OR (("cell respiration"[MeSH Terms] OR ("cell"[All Fields] AND "respiration"[All Fields]) OR "cell respiration"[All Fields] OR "oxygenation"[All Fields] OR "oxygen"[MeSH Terms] OR "oxygen"[All Fields] OR "oxygen s"[All Fields] OR "oxygenate"[All Fields] OR "oxygenated"[All Fields] OR "oxygenates"[All Fields] OR "oxygenating"[All Fields] OR "oxygenations"[All Fields] OR "oxygenative"[All Fields] OR "oxygenator s"[All Fields] OR "oxygenators"[MeSH Terms] OR "oxygenators"[All Fields] OR "oxygenator"[All Fields] OR "oxygene"[All Fields] OR "oxygenic"[All Fields] OR "oxygenous"[All Fields] OR "oxygens"[All Fields]) AND ("target"[All Fields] OR "targetability"[All Fields] OR "targetable"[All Fields] OR "targeted"[All Fields] OR "targeting"[All Fields] OR "targetings"[All Fields] OR "targets"[All Fields] OR "targetted"[All Fields] OR "targetting"[All Fields]))) AND ("heart arrest"[MeSH Terms] OR ("heart"[All Fields] AND "arrest"[All Fields]) OR "heart arrest"[All Fields] OR ("cardiac"[All Fields] AND "arrest"[All Fields]) OR "cardiac arrest"[All Fields] OR "OHCA"[All Fields] OR ("out of hospital cardiac arrest"[MeSH Terms] OR ("out of hospital"[All Fields] AND "cardiac"[All Fields] AND "arrest"[All Fields]) OR "out of hospital cardiac arrest"[All Fields] OR ("out"[All Fields] AND "hospital"[All Fields] AND "cardiac"[All Fields] AND "arrest"[All Fields]) OR "out of hospital cardiac arrest"[All Fields]))

*Web of Science*

ALL=((<hyperoxia> OR <hyperoxemia> OR <hyperoxygenation> OR <overoxygenation> OR <normoxia> OR <normoxemia> OR <permissive hypoxia> OR <permissive hypoxemia> OR <oxygenation targets>) AND (<cardiac arrest> OR <OHCA> OR <out-of-hospital cardiac arrest>))

*Cochrane Library*

(<hyperoxia> OR <hyperoxemia> OR <hyperoxygenation> OR <overoxygenation> OR <normoxia> OR <normoxemia> OR <permissive hypoxia> OR <permissive hypoxemia> OR <oxygenation targets>) AND (<cardiac arrest> OR <OHCA> OR <out-of-hospital cardiac arrest>), all field

**Figure S1: Survival to ICU discharge**


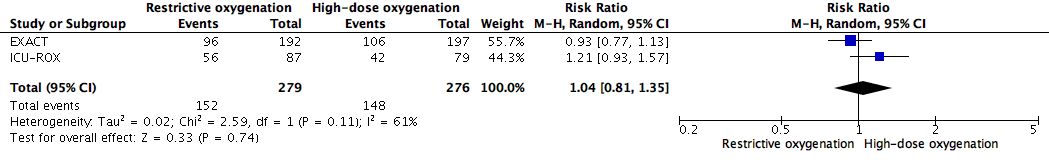


**Figure S2: Favorable neurological outcome at discharge**


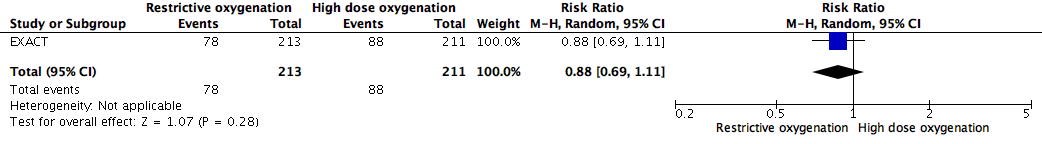


**Figure S3: Subgroup analysis of primary outcome: oxygenation targets**


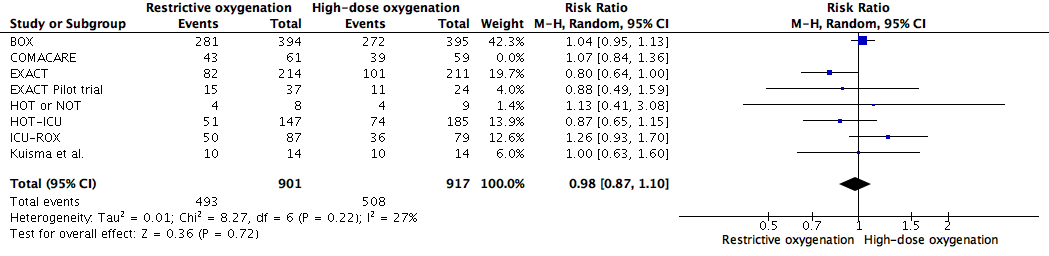


**Figure S4: Subgroup analysis of primary outcome: pre-hospital trials**


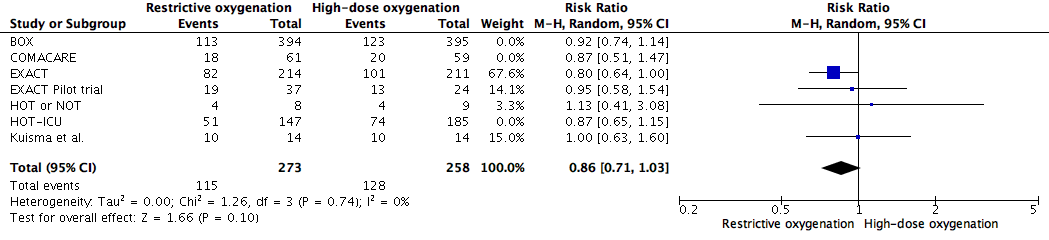


**Figure S5: Sensitivity analysis of primary outcome**


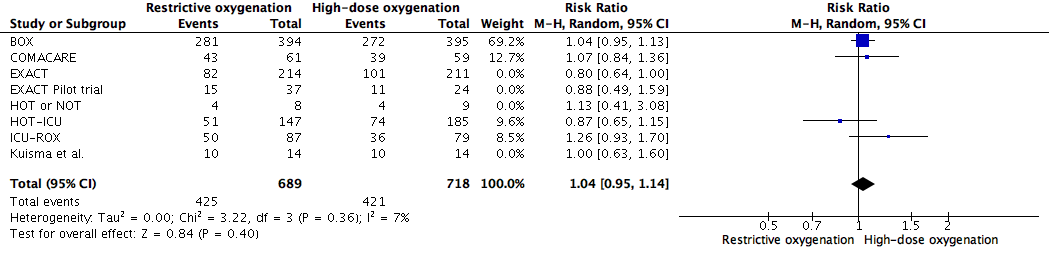


**Table S1: Oxygenation status on trial level**

|  | | **Kuisma et al.**  **2006** | **HOT or NOT**  **2014** | **EXACT PILOT 2018** | **COMACARE**  **2018** | **ICU-ROX**  **2020** | **BOX**  **2022** | **EXACT**  **2022** | **HOT-ICU**  **2023** |
| --- | --- | --- | --- | --- | --- | --- | --- | --- | --- |
| Restrictive group | Median PaO2, *kPa* | - // 14.6 | 9.7 // 10.7# | - // - | - // - | 14.2 // 11.2 | 16.1 // - | - // 8.0 | 11 // 9.8 |
| High-dose group | Median PaO2, *kPa* | - // 46.5 | 14.3 // 10# | - // - | - // - | 12.6 // 11.7 | 17.1 // - | - // 8.0 | 11.3 // 12.7 |
| Restrictive group | Median SaO2, *Sp02,* % | - // 98.3 | 96 // 96#, *94* | - // *97** | - // - | *98* // - | - // - | *99* // *97* | 95 // 93 |
| High-dose group | Median SaO2, *Sp02,* % | - // 99.5 | 99 // 97#, *99* | - *// 99** | - // - | *96* // - | - // - | *99* // *99* | 95 // 96 |
| Restrictive group | Median FiO2 | 0.3 // 0.33 | - // - | - // - | - // 0.35 | 0.64 // - | - // - | - // 0.5 | 0.7 // 0.39 |
| High-dose group | Median FiO2 | 1.0 // 1.0 | - // - | - // - | - // 0.5 | 0.64 // - | - // - | - // 0.8 | 0.7 // 0.53 |
| Legend:  Oxygenation at baseline // during intervention;  -: not (separately) reported; FiO2: fraction of inspired oxygen; PaO2: partial pressure of arterial oxygen; SaO2: oxygen saturation measured in blood gas analysis; Sp02: oxygenation saturation calculated from pulse oximetry; *: mean instead of median; #: t=24h of 72h | | | | | | | | | |

**Table S2: Event data on efficacy and safety outcomes**

|  | | | | | | | | | |
| --- | --- | --- | --- | --- | --- | --- | --- | --- | --- |
|  | | **Kuisma et al.**  **2006** | **HOT or NOT**  **2014** | **EXACT PILOT 2018** | **COMACARE**  **2018** | **ICU-ROX**  **2020** | **BOX**  **2022** | **EXACT**  **2022** | **HOT-ICU**  **2023** |
| Short-term survival (up to 90 d) | Restrictive group | 10/14 (h) | 4/8 (h) | 19/37 (h) | 18/61 (30 d) | 50/87  (h) | 281/394 (90 d) | 82/214 (h) | 51/147 (90 d) |
| Short-term survival (up to 90 d) | High-dose group | 10/14 (h) | 4/9 (h) | 13/24 (h) | 20/59 (30 d) | 36/79  (h) | 272/395 (90d) | 101/211 (h) | 74/185 (90 d) |
| Survival to hospital discharge | Restrictive group | 10/14 | 4/8 | 19/37 | - | 50/87 | - | 82/214 | - |
| Survival to hospital discharge | High-dose group | 10/14 | 4/9 | 13/24 | - | 36/79 | - | 101/211 | - |
| Survival to ICU discharge | Restrictive group | - | - | - | - | 56/87 | - | 96/192 | - |
| Survival to ICU discharge | High-dose group | - | - | - | - | 42/79 | - | 106/197 | - |
| Favorable neurological outcome (CPC ≤2) at discharge | Restrictive group | - | - | - | - | - | - | 78/213 | - |
| Favorable neurological outcome (CPC ≤2) at discharge | High-dose group | - | - | - | - | - | - | 88/211 | - |
| Desaturation/Hypoxia, patients | Restrictive group | 0/14 (not defined) | 7/8 (<88%) | 7/37 (<90%) | - | - | - | 67/214 (<90%) | - |
| Desaturation/Hypoxia, patients | High-dose group | 0/14 (not defined) | 3/9 (<88%) | 1/24 (<90%) | - | - | - | 34/211 (<90%) | - |
| Legend:  -: not (separately) reported; h: hour; d: days; CPC: cerebral performance category; ICU: intensive care unit; SAE: serious adverse event; TTM: target temperature management; QoL: quality of life; (h): in-hospital period; (30 d) within 30 days; (90 d) within 90 days | | | | | | | | | |
